# Adherence to a Mediterranean Diet and Lifestyle and Associations with Global Endometriosis Symptoms: A Cross-Sectional Analysis of Australian Women

**DOI:** 10.3390/nu18213544

**Published:** 2026-10-27

**Authors:** Courtney Harris, Anthony Villani, Nicole Scannell, Nina A. Wilson, Evangeline Mantzioris

**Affiliations:** 1 School of Health, University of the Sunshine Coast, Sippy Downs, QLD 4556, Australia; courtneymh19@gmail.com (C.H.); nicole.scannell@research.usc.edu.au (N.S.); 2 School of Allied Health and Human Performance, College of Health, Adelaide University, Adelaide, SA 5000, Australia; anthony.villani@adelaide.edu.au (A.V.); nina.wilson@adelaide.edu.au (N.A.W.); 3 Alliance for Research in Exercise, Nutrition and Activity (ARENA), College of Health, Adelaide University, Adelaide, SA 5000, Australia

**Keywords:** Mediterranean Diet, lifestyle, endometriosis, endometriosis symptoms, women’s health

## Abstract

Background/Objectives: Adherence to a Mediterranean diet (MedDiet) has been shown to improve women’s reproductive health. However, there is a lack of evidence on the potential efficacy in attenuating the intensity of global symptoms related to endometriosis. This was a cross-sectional analysis investigating the association between MedDiet (and associated lifestyle) adherence and intensity of global endometriosis symptoms in Australian women. Methods: Adherence to a MedDiet (and associated lifestyle) was assessed using the Mediterranean Diet Adherence Screener (MEDAS) and MEDiterranean LIFEstyle Index (MEDLIFE), and the impact of endometriosis symptoms was assessed using the Endometriosis Impact Questionnaire (EIQ-63). Results: A total of *n* = 103 participants (29.7 ± 8.6 years; BMI: 27.1 ± 6.8 kg/m^2^) were included in the final analyses. Participants reported moderate MEDLIFE adherence scores (12.1 ± 3.2) and low-to-moderate adherence to a MedDiet (5.6 ± 2.0). Few participants achieved normative serve size criteria for core dietary constituents of a MedDiet, including legumes (*n* = 14, 16.7%), fruit (*n* = 11, 13.1%), fish and seafood (*n* = 9, 10.7%), nuts (*n* = 15, 17.9%) and olive oil (*n* = 2, 2.4%). MEDLIFE index or MEDAS adherence were not associated with global impact symptoms of endometriosis. Conclusions: Despite the null findings, these results provide an important contribution to the existing literature, highlighting the importance of diet quality on women’s health more broadly. Exploration of these findings using well-controlled longitudinal analyses and robust clinical trials is needed to discover causal relationships.

## 1. Introduction

Endometriosis is a chronic, estrogen-dominant, inflammatory condition [1] that affects approximately ~11.4% of Australian women [2,3], with women of maternal age [30–34 years] most likely to receive a diagnosis (3). The condition is characterised by the presence of endometrial-like tissue found ectopically within the uterine cavity, often resulting in chronic pelvic pain [4]. In addition, women may also experience endometriotic lesions on the peritoneal surface of organs, such as the bowel, uterus, ovaries, and/or bladder, on the lining of the peritoneal cavity and/or the pouch of Douglas [4]. The repetitive deposition of menstrual tissue and breakdown of established tissue subsequently results in persistent inflammation in the peritoneal cavity [4], whereby immune cells, pro-inflammatory cytokines and extracellular matrix metalloproteinases activate the peritoneal microenvironment, leading to differentiation, adhesion, proliferation and survival of ectopic cells [5].

Symptoms are heterogeneous and may include chronic abdominal pain, dysmenorrhea (period pain), dyspareunia (pain during sexual intercourse), bowel and bladder dysfunction, fatigue, anxiety, depression and/or infertility [4,6,7,8]. Despite the presence of these symptoms, many women are commonly undiagnosed due to various factors such as normalisation of abnormal menstruation, healthcare providers dismissing symptoms, lack of awareness and education, and social concealment of menstruation [9,10]. The burden of endometriosis is such that it negatively impacts all components of an individual’s quality of life as well as placing considerable strain on healthcare costs [11]. Specifically, endometriosis negatively impacts social activities [11], mental and psychological health [12], work and finances [11], increases absenteeism at work and/or school [11], and sexual relationships [13].

Current treatment and management strategies require a multidisciplinary approach and are dependent on patient presentation of symptoms, age, preferences, location of the disease and treatment costs [14]. While surgical approaches remain a viable option, their efficacy is compromised by factors such as postoperative recurrence and diminished ovarian reserve, thus impacting fertility [15,16]. Moreover, surgery is associated with long wait times and high out-of-pocket costs. In addition to surgical approaches, multimodal pharmacological treatment includes the use of analgesics, hormonal suppression, antidepressants, and anxiolytics [17]. First-line treatment typically involves the use of anti-inflammatory drugs (NSAID) alone or concurrently with hormonal contraceptives [18]. However, pharmacological approaches have been shown to have limited effectiveness [19] and are associated with severe side effects and high cessation rates [20]. Because of this, many women with endometriosis report using self-care or adopting lifestyle interventions as part of their self-management for symptom management [21]. In a cross-sectional analysis of Australian women with endometriosis (*n* = 484), Armour et al. [21] showed that non-pharmacological self-management strategies, such as meditation, rest, heat and use of cannabis or hemp/CBD oil, were highly prevalent to attenuate the intensity of symptoms. Moreover, there appears to be a strong interest in the use of complementary and alternative medicines (CAM) and nutritional supplements among women with endometriosis. Adamietz et al. [22] reported that women with endometriosis had a high interest in using CAMs and nutritional supplements but lacked adequate information about their efficacy; the investigators reported that exercise, vitamins, yoga, homoeopathy and trace elements were among the most frequently used. Similar findings have also been reported in the UK and Australia, with the use of combined multivitamin and mineral supplements, or individual nutrients, such as vitamin D, calcium and omega-3 fatty acids, being the most widely used to manage symptoms [23,24]. Despite this, the efficacy of nutritional supplements on management of endometriosis-related symptoms remains unclear. A previous systematic review and meta-analysis investigating dietary supplementation for managing endometriosis-associated pain showed a lack of efficacy for their use, largely due to significant methodological issues and heterogeneity in the studies investigated [25].

Another self-management approach amongst women with endometriosis is dietary reform [26]. There is some evidence suggesting that diet quality is poorer in women with endometriosis, with lower intake of vegetables, fruits, whole grains and omega-3 fatty acids, and higher intakes of red and processed meat and trans fats [27]. Nevertheless, this evidence is inconsistent. Within the Nurses’ Health Study II (NHSII), Dougan et al. [28] showed that adherence to a healthy dietary pattern (Alternative Healthy Eating Index) characterised by a high intake of fruits, vegetables, whole grains, nuts and legumes was inversely associated with an endometriosis diagnosis; in contrast, adherence to a Western dietary pattern, characterised by a high intake of red and processed meats and ultra-processed foods, was positively associated with the risk of an endometriosis diagnosis. Nevertheless, a key gap in the literature is whether diet and nutrition can attenuate the intensity of global symptoms and delay the progression of endometriosis. At present, there is a lack of evidence-based clinical guidelines on diet and nutrition in endometriosis. Because of the role of inflammation in endometriosis, the role of plant-based dietary patterns may offer alternative therapeutic benefits for attenuating symptoms. In this context, the MedDiet, often described as an anti-inflammatory dietary pattern, is characterised by a high consumption of fruits, vegetables, nuts, legumes, wholegrains and extra-virgin olive oil (EVOO); moderate consumption of fermented dairy, eggs and red wine (consumed with meals only); and a low and/or infrequent consumption of red meat and meat products, butter, vegetable oils and processed foods [29]. There is a large and consistent body of evidence to support the use of the MedDiet in a number of chronic conditions, including metabolic syndrome, diabetes, cardiovascular disease, and mental health disorders [30,31,32]. However, there is a paucity of evidence in endometriosis. Being predominantly plant-based, the MedDiet is low in saturated and trans fats, and rich in vitamins and minerals, carotenoids, unsaturated fatty acids, and phenolic compounds, all possessing antioxidant and anti-inflammatory benefits [33]. Therefore, the potential mechanistic benefit related to the MedDiet and attenuation of endometriosis-related symptoms may relate to the anti-inflammatory potential of the diet, reductions in oxidative stress and a higher intake of antioxidants and phytonutrients.

Therefore, this cross-sectional study investigated the association between adherence to a MedDiet, associated lifestyle factors, and global symptoms (physical, psychosocial, lifestyle and societal) related to endometriosis amongst Australian women.

## 2. Methods

### 2.1. Study Design, Setting and Participants

A cross-sectional online study was undertaken among female adults aged ≥18 years with endometriosis. Australian female adults who had a self-reported medical diagnosis of endometriosis, resided permanently in Australia, and could independently complete an online survey in English were eligible to participate. A study flyer and social media tile were created and shared on social media platforms, including Facebook, Instagram, LinkedIn, X (formerly Twitter), and Bluesky, seeking voluntary participation. Australian-based Endometriosis support groups and organisations were also contacted seeking voluntary participation. Participant recruitment occurred from 29 April 2025 to 1 January 2026. The online survey was created and distributed using Qualtrics^TM^ survey software, and the survey link was shared on the aforementioned social media platforms, including details regarding the study protocol and contact details of the research team. The study was conducted according to the guidelines outlined in the Declaration of Helsinki, and ethics approval was granted by the Human Research Ethics Committees at the University of the Sunshine Coast (S252066) and the University of South Australia (S206791), respectively. Prior to participation, all participants acknowledged and agreed to an informed consent statement. Lastly, the present study has been reported in accordance with the STROBE-nut checklist [34].

### 2.2. Data Collection

A 155-item self-administered online questionnaire was used to explore the relationship between adherence to the MedDiet (and associated lifestyle) and the global symptoms and the long-term impact of endometriosis on varying aspects of women’s lives. The questionnaire was divided into three separate sections, including two previously validated survey instruments: (1) the MEDiterranean LIFEstyle Index (MEDLIFE; 28-item Mediterranean Lifestyle Questionnaire) [35]; (2) the Endometriosis Impact Questionnaire (63-item EIQ) [36]. The questionnaire also included a range of open- and closed-ended questions related to participant demographic characteristics, which included self-reported weight and height, age, ethnicity, education and socioeconomic status, year of diagnosis, comorbidities and other health conditions, use of prescription medications (for any related medical condition) and nutritional supplements, smoking status, physical activity, and sleep quality. The questionnaire was designed to be completed in approximately 20–25 min; however, no time restrictions were implemented. The link to the survey was recognisable only once to the server it was sent to prevent duplication when responding to the survey. IP addresses and viewed postcodes of all participant responses were also screened independently by the investigators to ensure individual responses aligned with the inclusion criteria.

### 2.3. Mediterranean Diet and Lifestyle Adherence

Adherence to the MedDiet and associated lifestyle was assessed using the validated 14-item MEDAS tool and validated 28-item MEDLIFE Index. The MEDAS tool was used to assess principal dietary constituents and food intake behaviours consistent with a MedDiet, whereby each question was scored dichotomously as 0 or 1. The MEDLIFE Index was used to capture adherence to an overall Mediterranean healthy lifestyle, inclusive of non-nutritional aspects, including physical activity, rest, social habits, conviviality and healthy culinary techniques [35]. Responses varied, with questions asking participants to either respond with a yes/no response, the frequency of times per week they would consume a specific food or food group, or the quantity of minutes-to-hours they would conduct certain lifestyle behaviours each day or week. The MEDLIFE questions were also scored dichotomously as 0 or 1. Collectively, adherence to a MedDiet and lifestyle was scored out of 27 (MEDLIFE Index combined with MEDAS), with a higher score reflecting greater adherence to a MedDiet and lifestyle. A MEDAS score greater than 10 is categorised as high adherence, a score between 6 and 9 reflects moderate adherence, and a score less than 5 represents low adherence.

### 2.4. Impact of Endometriosis

The long-term impact of endometriosis on varying aspects of women’s lives was assessed using the previously validated 63-item EIQ. The survey instrument was designed to measure the global impact of endometriosis and the impact of symptoms over 8 individual dimensions: physical, psychological, social, sexual and intimate relationships, fertility, employment and financial, educational and lifestyle. The questionnaire asks participants how endometriosis has affected their lives over three recall periods: the previous 12 months; the previous 1 to 5 years; more than 5 years ago. All EIQ items were ranked using a 5-point Likert scale, with each item contributing equally. To assess the impact of endometriosis within each dimension (e.g., physical impact), the ranked responses from each item within a recall period (e.g., last 12 months) were summed and rescaled to 0–100 in accordance with standard scoring procedures [36]. This process was repeated for the remaining two recall periods. The average of the three recall periods was subsequently calculated to provide the overall impact of endometriosis within that dimension. To calculate the global impact of endometriosis, the average of all dimensions was calculated. The global and individual dimensions were rescaled to 0–100, with higher scores reflective of a greater impact of symptoms.

### 2.5. Statistical Analysis

All continuous variables are presented as mean ± standard deviation (SD), or median and interquartile ranges (IQR). Categorical data are presented as frequencies and percentages. Multiple regression diagnostics were performed prior to regression analyses to ensure assumptions of multicollinearity and homoscedasticity were not violated. Univariable and multivariable linear regression analyses (and 95% confidence intervals (CIs)) were used to investigate the independent association between adherence to a Mediterranean Diet and Lifestyle (MEDLIFE Index), the global impact of endometriosis, and all individual dimensions of endometriosis symptoms across all three recall periods, using one unadjusted and four adjusted models. Univariable and multivariable regression analyses were also applied to adherence to a MedDiet (MEDAS only) to explore the independent relationship between diet and the global impact of endometriosis and all individual dimensions of endometriosis. Covariates used in the predictor models included age, BMI, smoking status, and active years with endometriosis. Analyses were performed using Statistical Package for the Social Sciences (SPSS) for Mac OS 31.0 software (IBM Corp., Armonk, NY, USA), with statistical significance set at *p* < 0.05.

## 3. Results

A total of *n =* 103 women participated in the online questionnaire, of which *n =* 103 participants (29.7 ± 8.6 years; BMI: 27.1 ± 6.7 kg/m^2^) met the inclusion criteria and were included in the final analyses. The majority of participants were born in Australia (*n* = 87; 84.5%); however a variety of cultural backgrounds were reported, with the two most prominent being English (*n* = 53, 51.5%) and Australian (*n* = 17, 16.5%). Two participants (2.8%) identified as Indigenous Australians. Over three-quarters (*n* = 80, 77.7%) of participants reported taking one or more prescription medications, with contraceptive (*n* = 44, 42.7%), anti-depressant (*n* = 28, 27.2%) and cardiovascular agents (*n =* 12, 11.7%) being the three most frequently reported. Over half of participants (*n* = 55, 53.9%) also reported taking one or more nutritional supplements and/or complementary medicines, with magnesium (*n* = 19, 18.4%), vitamin D (n = 17, 16.5%) and Iron (n = 15, 14.6%) being the three most frequently reported. Additionally, the three most frequent medical conditions reported by participants included anxiety (*n* = 54, 52.4%), depression (*n* = 35, 34.0%) and Asthma (*n* = 27, 26.2%). Additional demographic characteristics are presented in Table 1.

Overall, participants reported low-to-moderate adherence to a MedDiet (5.6 ± 2.0; range: 2–11). Few participants reported achieving normative serve size criteria for core dietary components of the MEDAS, including legumes (*n* = 14, 16.7%), fruit (*n* = 11, 13.1%), fish and seafood (*n* = 9, 10.7%), nuts (*n* = 15, 17.9%) and olive oil (*n* = 2, 2.4%). Nonetheless, almost half of participants (*n* = 50, 48.5%) reported using olive oil as their main source of dietary fat, over half reported preferentially consuming white meat over red meat (*n* = 71, 68.9%) and over half reported achieving normative serve size criteria for vegetables (*n* = 63, 61.2%). The proportion of participants achieving normative criteria for each individual component of the MEDAS is presented in Table 2.

Participants reported moderate adherence to a Mediterranean-style diet and lifestyle (MEDLIFE Index) (12.1 ± 3.2). Through analysis of the additional MEDLIFE questions, over half (*n* = 69, 67.0%) of participants reported preferentially consuming seasonal and traditional local products and fresh, minimally processed foods; half of participants (*n =* 52, 50.5%) reported usually eating in the company of family, friends or colleagues; and half (*n* = 52, 50.5%) reported preferring to consume foods in moderation by trying to choose smaller portion sizes. However, only a third (n = 30, 29.1%) of participants reported spending >20 min having lunch during weekdays.

According to EIQ-63 scores, the total sample reported a moderate global impact of endometriosis (49.2 (20.7)). Impact of endometriosis across individual dimensions varied among participants, with the social dimension representing the highest individual impact (69.8 (50.5)) and the lifestyle dimension representing the lowest impact (11.8 (28.5)). The EIQ-63 scores for all dimensions are presented in Table 1.

Adherence to a MedDiet and lifestyle (MEDLIFE Index) and the global impact of endometriosis and impact across individual dimensions are outlined in Table 3. Our analysis showed that adherence to the MEDLIFE index was not associated with the global impact of endometriosis. Moreover, adherence to the MEDLIFE index as well as individual dietary constituents of the MedDiet (when assessed against MEDAS) was not related to individual impact dimensions of endometriosis.

## 4. Discussion

The primary aim of this cross-sectional study was to explore the association between adherence to a MedDiet and lifestyle and global symptoms of endometriosis amongst Australian women. We observed that adherence to a MedDiet and lifestyle (as defined by the MEDLIFE Index and MEDAS) was not related to the global impact of endometriosis (as assessed by the EIQ). Nevertheless, our study was limited by a small sample size and a lack of high adherence to a MedDiet within the study cohort.

To the best of our knowledge, few studies have investigated the relationship between MedDiet adherence and the global symptoms related to endometriosis. In a previous prospective study of *n* = 35 Italian women with endometriosis, Cirillo et al. [37] reported that MedDiet adherence was inversely associated with non-menstrual pelvic chronic pain, dyspareunia, dysuria and dyschezia at three months, and dyschezia and dyspareunia at 6 months. These results suggest that following a healthy dietary pattern, such as the MedDiet, could present as a novel therapeutic lifestyle approach for pain management in endometriosis. However, unlike the aforementioned study, we assessed the intensity and impact of global endometriosis symptoms, rather than just pain. Moreover, the study by Cirillo et al. [37] assessed MedDiet adherence using the MEDI-LITE adherence tool [38], and adherence scores were greater [37], which is not surprising given the differences in geographical location and culture (Australian vs. Italian) between the two study groups. Importantly, due to differences in scoring protocols, MedDiet adherence tools are not comparable, making assessments between studies challenging. Nevertheless, our null findings, in contrast to the aforementioned study may be related to the lack of high adherence scores, reflecting the difficulties of adopting a MedDiet in non-Mediterranean countries. In a cross-sectional analysis of *n * =  606 Australian adults, our group previously reported multiple barriers towards adherence and uptake of a MedDiet, including knowledge, motivation, affordability, time, and suitability [39]. At present, there is limited literature on the feasibility and acceptability of following a MedDiet in Australian women with endometriosis; as such, it is unknown whether there are specific challenges associated with uptake within this population of women. However, in a similar cohort of Australian women, Scannell et al. [40,41] reported that a 12-week MedDiet intervention was acceptable and feasible amongst overweight women with polyendocrine metabolic ovarian syndrome (PMOS).

The proposed mechanistic benefits of the MedDiet and attenuation of symptoms related to endometriosis are attributed to its anti-inflammatory properties; nonetheless, to date, no studies have evaluated the therapeutic potential of a MedDiet intervention using well-designed and robust clinical trials in patients with already diagnosed endometriosis. In a single-arm study of *n* = 68 women from Austria, Ott et al. [42] reported that following a MedDiet significantly reduced or improved general pain, dysmenorrhea, dyspareunia, and dyschezia. Despite the positive outcomes for endometriosis symptoms, importantly, this was an uncontrolled study, and therefore it remains unknown whether these benefits were attributable to the dietary intervention. However, certain dietary constituents of a MedDiet, such as fish and extra-virgin olive oil, have known anti-inflammatory properties, which may be of benefit. Specifically, omega-3 fatty acids have been shown to reduce pro-inflammatory cytokines (TNF-alpha, IL-6 and IL-1) in patients with endometriosis; however, a recent meta-analysis by Liu et al. [43] showed that omega-3 fatty acids did not significantly alleviate endometriosis-related symptoms related to pain, sexual dysfunction, or quality of life. Extra virgin olive oil, which contains phenolic compounds, displays a similar structure to the molecule ibuprofen, and both possess anti-inflammatory effects via the same mechanism, i.e., cyclooxygenase inhibition [44]. Nevertheless, there is little direct evidence linking the benefits of extra-virgin olive oil with symptom reduction in endometriosis.

Many women with endometriosis employ self-management strategies, including diet, to mitigate symptoms and improve their health-related quality of life [45]. Whilst it is acknowledged that diet and nutrition can play a key role in symptom management [26], at the present time, the benefits specifically related to the MedDiet are theoretical and lack clinical evidence for endometriosis [44,46,47]. Several dietary (e.g., low FODMAP, gluten-free, anti-inflammatory, high-fibre) and nutritional approaches have been investigated in patients with endometriosis [26,48]; however, there is insufficient evidence supporting the use of a single dietary or nutritional approach as an adjuvant therapy for endometriosis. As such, patients with endometriosis will likely benefit from a personalised dietary approach, which considers a range of clinical and lifestyle history [26]. Although there is evidence of poor diet quality in women with endometriosis [27], there is also evidence to support that women make positive dietary choices following a diagnosis of endometriosis (increased consumption of fruits, vegetables, whole grains, legumes and fish), suggesting women are interested in adopting dietary change to support symptom management [49]. In our analysis, few participants achieved normative serve size criteria for key food groups consistent with a traditional MedDiet, including nuts (16.9%), legumes (14.1%), fish and seafood (12.7%), fruit (8.5%) and olive oil (2.8%), suggesting a proxy measure of poor diet quality; nevertheless, given the cross-sectional nature of this research, it is unknown whether women changed their diet following a diagnosis.

Although we observed no relationship between MedDiet and lifestyle adherence and the global impact of endometriosis symptoms, following a MedDiet may still provide broad health benefits. There is supportive evidence to suggest that following a MedDiet may be beneficial for women’s reproductive health, including premenstrual syndrome, dysmenorrhea, sexual dysfunction, endometriosis, menopause, PMOS, and infertility [50,51,52,53,54]. Although further prospective studies and robust clinical trials are needed, individualised medical nutrition therapy coupled with the adoption of healthy dietary patterns, such as the MedDiet, to mitigate the global symptoms associated with endometriosis should be considered.

Several limitations should be considered when interpreting the findings of our study. Firstly, the cross-sectional study design limits the ability to establish causation. Secondly, due to recruitment methods using social media for the online survey, selection bias was highly possible, which may restrict the extent to which our findings can be generalised to the broader population of women with endometriosis, particularly those unable to participate in an online survey or have access to social media. Furthermore, our sample was also predominantly younger and highly educated; of note, there is evidence to support that highly educated females are more likely to respond to social media surveys [55]. Additionally, the use of self-reported instruments to capture adherence to a MedDiet and lifestyle and the impact of endometriosis increases the potential for social desirability and/or recall bias. Secondly, women self-reported their diagnosis. As indicated, there was a lack of high adherers to a MedDiet; as such, our null findings may be related to an unrepresentative sample, rather than a lack of association with the diet. Moreover, our analysis did not consider or assess all aspects of dietary intake (e.g., total energy, macro- or micro-nutrient intake), with the use of a validated food frequency questionnaire.

## 5. Conclusions

This cross-sectional analysis showed that adherence to a MedDiet and lifestyle was not associated with global symptoms of endometriosis in this group of women with low adherence levels. However, evidence on the relationship between nutrition and diet and symptoms of endometriosis is scarce. As such, our results provide an important contribution to the existing evidence on the importance of diet quality and disease of the female reproductive system, and women’s health more broadly. Exploration of these findings using well-controlled longitudinal analyses and robust clinical trials, using biochemical markers of disease, such as inflammation, is needed to better elucidate these findings and discover causal relationships.

## Figures and Tables

**Table 1:** Participant demographic characteristics.

Characteristics	*n*	%
**Age** (years) Mean, SD	29.7	8.6
**BMI** (kg/m^2^) Mean, SD	27.1	6.8
**Education status** *n*, *%*		
Secondary school	26	25.2
Trade/technical/vocational training	10	9.7
Diploma	14	13.6
Bachelor’s degree	30	29.1
Postgraduate degree or doctorate	22	21.4
Did not disclose	1	1.0
**Total annual household income (before tax)** *n*, %		
$0–24,999	5	4.9
$25,000–49,999	9	8.7
$50,000–74,999	18	17.5
$75,000–99,999	12	11.7
$100,000–124,999	11	10.7
$125,000–149,999	8	7.8
$150,000–174,999	10	9.7
$175,000–199,999	11	10.7
$200,000 or greater	8	7.8
Did not disclose	11	10.7
**Use of prescription medicines** *n*,%	80	77.7
**Use of nutritional supplements or complementary medicines** *n*, %	55	53.4
**Active years with endometriosis (years)** Mean, SD	5.0	4.7
**14-item Mediterranean Diet Adherence (MEDAS)** Mean, SD	5.6	2.0
Mediterranean Lifestyle Index (MEDLIFE) * Mean, SD	6.6	1.8
**Combined MEDAS and MEDLIFE (score out of 27)** Mean, SD	12.2	3.2
**EIQ-63 Global score** Median, IQR	49.2	20.7
**EIQ-63 individual dimension scores** Median, IQR		
Physical	64.7	15.5
Psychological	69.3	36.0
Social	69.8	50.5
Sexual and intimate	47.0	48.8
Fertility	33.3	32.0
Employment	42.4	34.1
Educational	28.1	46.2
Lifestyle	11.8	28.5
**Smoking status** *n*, %		
Current smoker	6	5.8
Former smoker	10	9.7
Non-smoker	75	72.8
Did not disclose	12	11.7
**Physical activity duration** *n,* %		
<30 min	13	12.6
30–59 min	38	36.9
60–89 min	16	15.5
90–119 min	4	3.9
≥120 min	18	17.5
Did not disclose	14	13.6
**Sedentary activity (hours/day)** *n*, %		
<2 h	2	1.9
2–3 h 59 min	11	10.7
4–5 h 59 min	20	19.4
6–7 h 59 min	21	20.4
8–9 h 59 min	16	15.5
≥10 h	20	19.4
Did not disclose	13	12.6
**Sleep quality** *n*, %		
Very good	4	3.9
Fairly good	51	49.5
Fairly bad	32	31.1
Very bad	7	6.8
Did not disclose	9	8.7

SD, Standard Deviation; IQR, Inter Quartile Range; BMI, Body Mass Index; EIQ-63, 63-item Endometriosis Impact Questionnaire. * Block 2 and 3 questions from MEDLIFE Index.

**Table 2:** Proportion of participants achieving normative criteria for each individual MEDAS question.

MedDiet Food Components, Achieved	*n*	%
Olive oil as main dietary fat source	50	48.5
Olive oil serves	2	1.9
Vegetable serves	63	61.2
Fruit serves	11	10.7
Infrequent red and processed meats	30	29.1
Infrequent butter, margarine, cream	28	27.2
Infrequent sweet, carbonated beverages	48	46.6
Red wine consumption	1	1.0
Legume serves	14	13.6
Fish and seafood serves	9	8.7
Infrequent commercial sweets and pastries	62	60.2
Nut serves	15	14.6
White meat preference over red meat	71	68.9
Consumption of tomato-based dishes	58	56.3

**Table 3:** Linear regression coefficients (and 95% CI) expressing independent associations between adherence to a Mediterranean-style diet and lifestyle and the impact of endometriosis across each dimension *.

EIQ-63 Dimensions of Endometriosis	Beta *	95% CI	*p*
Global impact	−0.069	−0.050, 0.027	0.560
Physical impact	−0.042	−0.042, 0.029	0.725
Psychological impact	−0.124	−0.049, 0.016	0.307
Social impact	−0.208	−0.049, −0.004	0.089
Sexual and intimate impact	−0.160	−0.056, 0.013	0.214
Fertility impact	0.172	−0.014, 0.054	0.242
Employment and financial impact	−0.127	−0.049, 0.016	0.305
Educational impact	0.064	−0.024, 0.037	0.665
Lifestyle impact	−0.009	−0.040, 0.037	0.943

Beta, standardised beta coefficient; CI, Confidence Interval; EIQ-63, Endometriosis Impact Questionnaire. * Standardised beta coefficient represents the change in SD-unit increase in the Mediterranean Diet and Lifestyle score per change in outcome measure.

## Data Availability

Data that support these findings is available from the corresponding author upon reasonable request.
